# Patient and Provider Perspectives of a Web-Based Intervention to Support Symptom Management After Radioactive Iodine Treatment for Differentiated Thyroid Cancer: Qualitative Study

**DOI:** 10.2196/60588

**Published:** 2025-03-19

**Authors:** Alaina L Carr, Angela M Jenkins, Jacqueline Jonklaas, Kate Gabriel, Kristen E Miller, Kristi D Graves

**Affiliations:** 1 Department of Oncology, Lombardi Comprehensive Cancer Center, Georgetown University, Washington, DC, United States; 2 Department of Natural Sciences, Bowie State University College of Arts and Science, Bowie, MD, United States; 3 Division of Endocrinology, Georgetown University, Washington, DC, United States; 4 Department of Health Systems Administration, Georgetown University School of Health, Washington, DC, United States; 5 MedStar Health Research Institute, National Center for Human Factors in Healthcare, Washington, DC, United States

**Keywords:** iodine radioisotopes, person-based approach, self-management, Social Cognitive Theory, survivorship, symptom burden, thyroid neoplasms, web-based intervention, radioactive iodine treatment, radiotherapy, thyroid cancer, qualitative, quality of life, survivorship care, supportive care, patient with cancer, QoL, cancer, carcinoma, malignancy, tumor, malignant, oncology, neoplasm, benign, neoplasia, thyroid

## Abstract

**Background:**

Patients diagnosed with differentiated thyroid cancer (DTC) who receive radioactive iodine (RAI) treatment experience acute, medium, and late treatment effects. The timing and severity of these effects vary by individual; common posttreatment effects include dry mouth, salivary gland swelling, dry eyes, and nose bleeds. The nature of symptoms that patients experience after RAI treatment can significantly and negatively impact health-related quality of life. Adequate information during the postprimary treatment phase remains an unmet need among the population of patients diagnosed with DTC.

**Objective:**

This qualitative study aimed to identify and understand self-management strategies for RAI-specific symptom burden from the perspectives of patients and stakeholders (cancer care providers and patient advocates). An additional aim included assessing features and functionalities desirable in the development of a web-based intervention to engage patients in their self-management and thyroid cancer survivorship care.

**Methods:**

Following the Social Cognitive Theory framework and person-based principles, we conducted six focus groups with 22 patients diagnosed with DTC who completed RAI treatment and individual interviews with 12 stakeholders in DTC care. The interviews focused on participants’ perspectives on current self-management strategies and mockups of a symptom management web-based intervention. Before focus groups and interviews, participants completed a demographics survey. Focus group discussions and interviews were transcribed and coded using content analysis. Interrater reliability was satisfactory (ɑ=.88).

**Results:**

A total of 34 individuals (patients and stakeholders) participated in the study; the mean age was 45 (SD 13.4) and 45.3 (SD 13) years, respectively. Three domains emerged from qualitative interviews: (1) difficult-to-manage RAI symptoms: short, medium, and late treatment effects; (2) key intervention structure and content feedback on mockups; and (3) intervention content to promote RAI symptom management and survivorship care. Focus group participants identified the most prevalent RAI symptoms that were difficult to manage as: dry mouth (11/22, 50%), salivary gland swelling (8/22, 36%), and changes in taste (12/22, 55%). Feedback elicited from both groups found education and symptom management mockup videos to be helpful in patient self-management of RAI symptoms, whereas patients and stakeholders provided mixed feedback on the benefits of a draft frequently asked questions page. Across focus groups and stakeholder interviews, nutrition-based symptom management strategies, communication with family members, and practical survivorship follow-up information emerged as helpful content to include in a future web-based supportive care intervention.

**Conclusions:**

Results suggest education and symptom management videos can empower patients with DTC to self-manage mild to moderate RAI symptoms on a web-based platform. Findings emphasized the need for additional information for patients related to ongoing care following RAI treatment including social support and thyroid cancer surveillance. The findings provide insights for theoretically informed interventions and recommendations for refinements in thyroid cancer survivorship from patient and provider perspectives.

## Introduction

### Background

Thyroid cancer is often mislabeled as the “good cancer” [1-3] due to the excellent 5-year survival rates compared to other cancers [4]. Differentiated thyroid cancer (DTC) is the most common endocrine malignancy among the estimated 44,020 new cases of thyroid cancer in the United States annually [4]. DTC involves a spectrum of disease presentations characterized by different levels of risk of recurrence. For patients with a high risk of recurrence, the standard of care involves total thyroidectomy followed by radioactive iodine (RAI) treatment and thyroid hormone therapy [5-7]. For patients with intermediate-risk disease, clinical practice guidelines from the American Thyroid Association recommend thyroidectomy followed by consideration of RAI treatment in consultation with their medical team [6]. Approximately, 72% of these intermediate and high-risk patients receive RAI and thyroid hormone therapy as standard of care [5]. Despite the favorable 5-year survival rates, complications from RAI treatment are common, impacting health-related quality of life (HRQOL) [1,8-10]. The “good cancer” label given to thyroid cancer frustrates patients, particularly when they experience side effects as a result of RAI treatment [1].

The likelihood of acute and chronic side effects following RAI varies based on RAI dose administration; these treatment impacts are particularly evident in patients who receive higher doses of ≥150 mCi [7,11,12]. While trends toward lower dose activity are recommended, therapeutic dose recommendations vary across professional medical societies and practice patterns [6,11-14]. Short-, medium-, and long-term complications from RAI treatment include salivary dysfunction, dysphagia, dysphonia, sialadenitis, appetite changes, nausea, vomiting, xerostomia, epiphora, and secondary malignancies [1,15-17], with the onset of certain symptoms appearing as late as 1-year post-RAI treatment [12,16]. In a cross-sectional study of 69 patients diagnosed with DTC and treated with RAI, lower HRQOL was significantly associated with higher symptom burden, distress, and lower self-efficacy [18]. Furthermore, patients with thyroid cancer report lower HRQOL compared to patients with other types of cancer with worse survival [10]. Additional care challenges reported by patients with DTC are unmet information needs about follow-up care, psychosocial support, and coordination of care [17,19]. Research is needed to understand, address, and mitigate RAI symptom burden among patients with DTC.

Mobile health (mHealth) technologies offer an accessible approach to symptom management interventions [20] and are increasingly used in the delivery of cancer care. mHealth refers to mobile and wireless technologies such as smartphones, computers, and tablets to support patient care, education, and communication in health care [21,22]. mHealth technologies have been implemented in the prevention, promotion, treatment, and maintenance of health and health care for patients with breast, lung, leukemia, and prostate cancers [21-25]. mHealth interventions empower patients with cancer with direct access to educational and support tools to improve self-management of symptoms, including fatigue, pain, distress, and cognitive impairment [21,23,26,27]. Core elements of mHealth interventions for chronic disease management include evidence-based education, monitoring and tracking symptoms, tailored feedback on symptoms, self-management training to cope with the psychological and physical aspects of treatment, and communication with providers [21,28,29]. Applying evidence-based theories to inform the design characteristics of digital technology interventions during the development phase of the research is one way to engage patients, foster patient-provider communication, and improve patient-centered health outcomes [20-22,28].

### Theoretically Driven mHealth Intervention Development

Social Cognitive Theory (SCT) [30,31], a longstanding theory-based approach to health behavior change, coupled with guiding principles from the person-based approach [20,32] to mHealth intervention development, offers a basis for core components of symptom management interventions. Behavioral interventions based on tenets of SCT are evaluated through patient knowledge of health risks and benefits, self-efficacy or confidence related to symptom management, outcome expectations about the costs and benefits of performing a particular behavior, and self-regulation impacting patient HRQOL [30,31]. Prior examples of the use of SCT in cancer-related health behavior change interventions include physical activity behavior change interventions [33-36] or interventions to promote HRQOL in breast cancer populations [37]. The application of SCT to promote self-management of RAI symptoms among patients with DTC is understudied, and further research is needed to identify core components of health-related interventions. The person-based approach to mHealth interventions yields insights into the behavioral components relevant to the populations intended to use the intervention [32]. As patients with DTC post-RAI treatment are commonly seen in ambulatory care settings, and their follow-up care is managed by specialists [17], a digital platform offers patients accessibility delivered at the point of need. Furthermore, involving target patient users and members of the health care team early in the intervention design process can identify key components [20,21,32] of symptom management interventions among this population.

We seek to develop and refine tailored mHealth symptom management interventions for patients with DTC who completed RAI. Thus, we conducted a qualitative study to identify and understand potential self-management strategies for RAI-specific symptoms from the perspectives of patients with DTC who completed RAI treatment and health care providers. In addition, we wanted to elicit feedback on core content and features in the formative development of a web-based intervention to promote patient self-management of RAI symptoms.

## Methods

### Study Design

We followed the COREQ (Consolidated Criteria for Reporting Qualitative Research) to report the qualitative findings [38]. From August 2022 to January 2024, we conducted a qualitative study using focus groups with patient participants and individual stakeholder interviews at Georgetown University Medical Center.

### Ethical Considerations

Ethics approval was performed in line with the principles of the Declaration of Helsinki. Approval was granted by the Georgetown University Institutional Review Board (#00005285). All focus group participants and stakeholders provided informed consent. Study data were deidentified prior to data analysis. Each participant received Amazon gift cards worth US $50 for their participation.

### Participant Selection and Setting

Adult patients were recruited from MedStar Georgetown University Hospital to take part in the qualitative study. Treating endocrinologists referred patients to the study. Patients were invited to participate if they were diagnosed with DTC, completed RAI treatment within the past three years, and had access to an electronic device with an internet connection. We excluded non-English-speaking patients and those with psychiatric or cognitive impairments that would inhibit meaningful consent.

For the stakeholder interviews, health care specialists were recruited from four hospitals in the Washington metropolitan area. Health care specialists were referred to the study by colleagues or invited to participate directly by study staff through an introductory email. Stakeholder inclusion criteria were self-identification as a full-time specialist treating patients with DTC post-RAI treatment or allied health professionals and patient advocates affiliated with ThyCa: Thyroid Cancer Survivors’ Association, Inc.

We used a purposive sampling strategy for patient age, gender, cancer type, and health subspecialists from academic and community hospital settings in the area. We recruited 22 patients diagnosed with DTC who completed RAI treatment and 12 stakeholders from local health care facilities and ThyCa groups in the DC area.

### Procedures and Data Collection

Following Institutional Review Board approval, study staff approached potential participants with an introductory study email followed by a phone call. Study staff completed informed consent through a 15-minute telephone or Health Insurance Portability and Accountability Act-compliant video conference and obtained electronic informed consent from focus group participants and stakeholders. Focus group participants and stakeholders completed a brief survey through REDCap (Research Electronic Data Capture; Vanderbilt University) [39] to obtain sociodemographic, clinical characteristics, and occupation information. The interviews were conducted over a Health Insurance Portability and Accountability Act-compliant videoconference lasting approximately two hours for focus group participants and one hour for individual stakeholder interviews.

Two authors (ALC: psychologist, female; and KDG: behavioral scientist, female) trained in qualitative research methods developed moderator and semistructured interview guides (MultimediaAppendices 1  2), which were informed by tenets of SCT [30] and person-based design principles [32]. Both guides included items to elicit participants’ perspectives about RAI symptoms that were challenging to manage, identify content that would be helpful in a symptom management website, and obtain feedback on the formatting and content of static images or mockups (Multimedia Appendix 3) of a prospective symptom management website. Participants were asked to provide feedback on the mockups after eliciting input on prospective content to minimize participant response bias. One author (ALC) conducted audio-recorded interviews for focus group and stakeholder interviews. A second member of the research team trained in qualitative research (KG: research assistant, female; Gautham Pillai: research assistant, male; and AMJ: research assistant, female) took field notes during the focus group and stakeholder interviews. All focus group and stakeholder interviews were transcribed verbatim and deidentified before data analysis. All focus group participants and stakeholders received a summary of the findings.

### Data Analysis

We used thematic content analysis to analyze focus group and stakeholder interview transcripts using a well-established 6-step framework [40]. Three authors (ALC, AMJ, and KG) independently reviewed transcripts to familiarize themselves with the data and identify initial codes using ATLAS.ti, a computer-assisted qualitative data analysis software (ATLAS.ti Scientific Software Development GmbH). The authors (ALC, AMJ, and KG) discussed initial codes, shared definitions of each code, and grouped the codes into broad, meaningful categories. The coders further organized the codes into major domains and subthemes and examined discrepant data until resolution. Analysis was systematically and iteratively applied across all transcripts. Thematic saturation [40] occurred after no new information was identified from transcripts. We calculated Krippendorf Cu-ɑ coefficients [41,42] to evaluate coding precision by the three team members for the three domains across transcripts. The final Krippendorf Cu-α=.88, indicating acceptable agreement [43]. Extracted quotations exemplified major domains and subthemes. Descriptive statistics were used to characterize focus group and stakeholder demographic, clinical, and occupation characteristics.

## Results

### Participants

**Figure 1.**
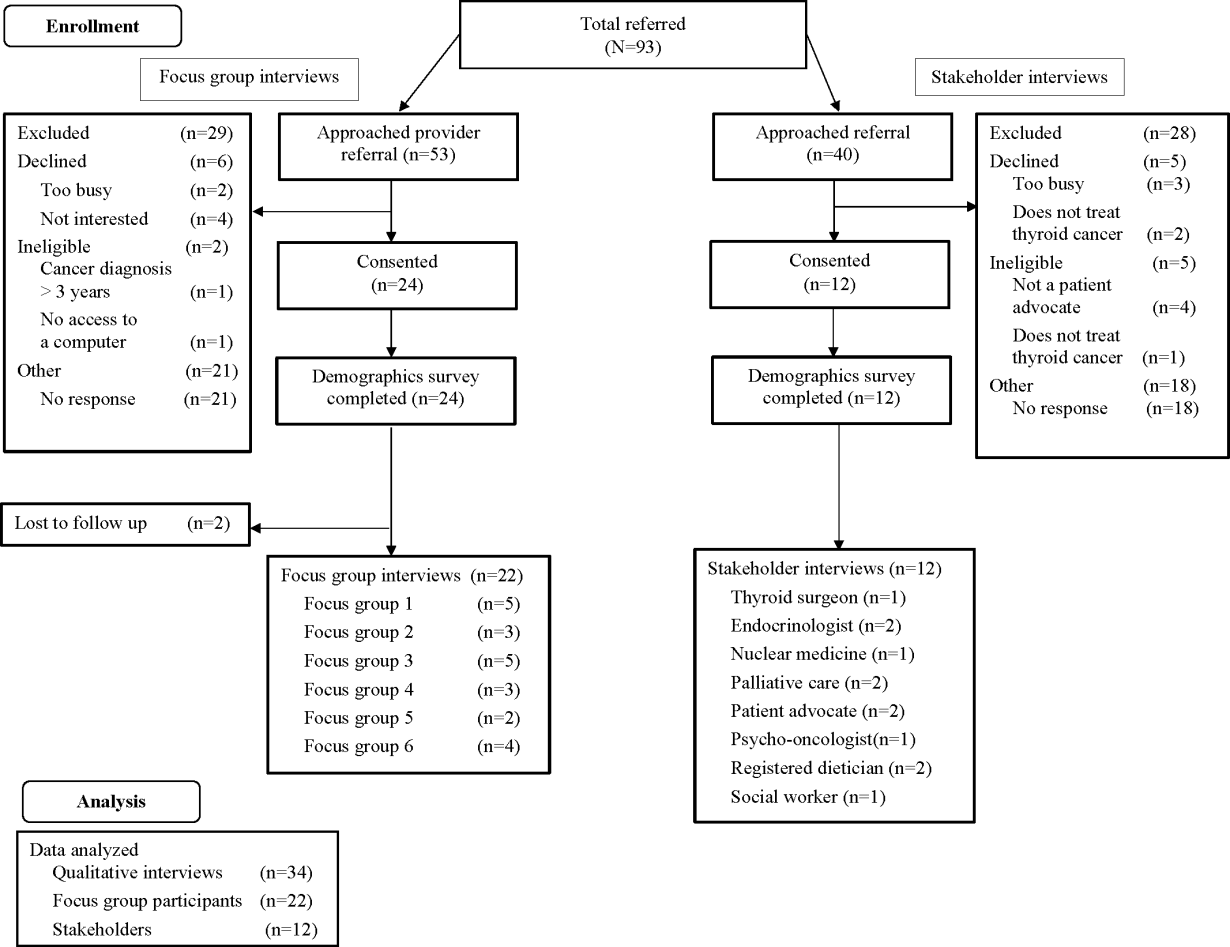
Qualitative study flowchart for focus group participants and stakeholders. Participants, recruited from clinical settings, were patients diagnosed with thyroid cancer. Stakeholders, recruited through professional networks, were providers involved in the clinical care of patients diagnosed with thyroid cancer. Data were collected between August 2022 and January 2024.

We approached 93 patients and stakeholders to enroll in the qualitative study; 24 patients and 12 stakeholders consented, 2 patients were unable to participate in focus groups due to scheduling conflicts, and 34 interviews were analyzed (Figure 1). A total of 29 patients were excluded due to ineligibility (n=2), decline (n=6), and no response to the introductory study email (n=21). A total of 28 stakeholders were excluded from the study due to ineligibility (n=5), decline (n=5), and no response to the introductory study email (n=18).

The age (in years) of focus group participants and stakeholders was 45.0 (SD 13.4; range 23.6‐72.5) and 45.2 (13.0; range 32.3‐75.7), respectively. The majority of focus group participants and stakeholders identified as female (15/22, 68%; 10/12, 83%), non-Hispanic or Latino (20/22, 91%; 11/12, 92%), and White (14/22, 64%; 8/12, 67%), respectively. In terms of clinical characteristics, the majority of focus group participants were diagnosed with papillary thyroid cancer (18/22, 82%). The average time since diagnosis (in years) was 1.5 (SD 1.0; range 0.03‐2.9), and the time since treatment (in years) was 1.1 (SD 0.9; range 0.1‐2.8). There was a total of six focus groups, with a range of 2‐5 participants in each group. Stakeholders consisted of thyroid surgeons (n=1), endocrinologists (n=2), nuclear medicine specialists (n=1), palliative care providers (n=2), patient advocates (n=2), psycho-oncologists (n=1), registered oncology dietitians (n=2), and social workers (n=1). Table 1 presents sample characteristics of focus group participants and stakeholders and Table 2 presents clinical characteristics of focus group participants.

**Table 1.** Sample characteristics of focus group participants and stakeholders (N=34)^a^.

Characteristics			Focus group participants(n=22)	Stakeholders(n=12)
**Age (years)**				
Mean (SD)			45.0 (13.4)	45.2 (13.0)
Range			23.6-72.5	32.3-75.7
Female, n (%)			15 (68)	10 (83)
Married or partnered, n (%)			14 (64)	—^b^
Hispanic or Latino, n (%)			2 (9)	1 (8)
**Race, n (%)**				
Asian			3 (14)	3 (25)
Black or African American			2 (9)	1 (8)
More than one race/ other			3 (14)	—
White			14 (64)	8 (67)
**Education, n (%)**				
High-school and below			3 (14)	—
College and above			19 (86)	—
**Employment status, n (%)**				
Full-time			17 (77)	—
Part-time			3 (14)	—
Student			2 (9)	—
Retired			2 (9)	—
Heath Insurance (Yes), n (%)			22 (100)	—

a Participants, recruited from clinical settings, were patients diagnosed with thyroid cancer. Stakeholders, recruited through professional networks, were providers involved in the clinical care of patients diagnosed with thyroid cancer. Data were collected between August 2022 and January 2024.

b Not applicable.

**Table 2.** Differentiated thyroid cancer clinical characteristics of focus group participants (n=22)^a^.

Clinical characteristics	Focus group participants
**Time since diagnosis (years)**	
Mean (SD)	1.5 (0.9)
Range	0.03‐2.9
**Time since treatment (years)**	
Mean (SD)	1.1 (0.9)
Range	0.10‐2.8
**Histology**^**b**^, n (%)	
Papillary	18 (82)
Follicular	2 (9)
Do not know	4 (18)
**Stage**^**b**^, n (%)	
I	6 (27)
II	2 (9)
III	1 (5)
IVA	1 (5)
Do not know	11 (50)

a Participants recruited from clinical settings were patients diagnosed with thyroid cancer. Data was collected between August 2022 and January 2024.

b The value given are frequencies.

### Qualitative Results

Qualitative results yielded three domains: (1) difficult-to-manage RAI symptoms: short, medium, and late treatment effects; (2) key intervention structure and content feedback on mockups; and (3) intervention content to promote RAI symptom management and survivorship care. Tables3  4 display exemplar quotes with patient identifiers to depict domains and subthemes from focus group participant and stakeholder interviews.

**Table 3.** Summary and exemplar quotes from focus group participants and stakeholders related to domain 2 key intervention structure and content feedback on mockups with an SCT^a^ framework.

Mockup	Exemplar quotes	SCT^a^ component
Home pageDescription: benefits and consequences home page in its current format; recommended changes to enhance patient understanding of website component	“In those websites you always see younger people. I think it’s nice to include more diversity. If you are going to include more pictures have other ones where you see people that are older depicted.” [Focus Group 5, PID 2] “On the landing page ... You can have a tab on statistics ... Are you experiencing side effects? Side effects management tab here. Social resources here. Or supportive care resources. You could have a separate nutrition tab.”‬ ‬‬‬‬‬‬‬‬‬‬[PID 10, Registered Oncology Dietitian]	Knowledge and outcome expectations
Education page RAI^b^ salivary symptomsDescription: benefits of education page on RAI symptom self-management; recommended changes to enhance engagement in website component	“[It] Feels friendly discussing something that is very distressing. There is an empowerment element to this of, ‘Hey, we get you. You’re experiencing these things. Please watch out for these and call your doctor.’ This page is magical. I wish this site existed already.”‬ [‬‬‬‬‬‬‬‬‬‬‬‬‬‬‬‬‬‬‬‬‬‬Focus Group 4, PID 3] “It’s a good general informational landing spot particularly things that they should definitely not try home remedies for. Like the bottom half [Redness over salivary glands, Pain doesn’t go away with antiinflammatory medications, worsening swelling or salivary glands-signs of infection, Significant weight loss due to difficulty eating and swallowing].” [PID 8, Thyroid Surgeon]	Knowledge
Symptom management videoDescription: benefits of RAI symptom self-management video on self-efficacy; consequences of self-management videos on self-efficacy; recommended changes to enhance patient engagement in self-management symptom videos	“Having this particular type of video on how to massage salivary glands, I would be interested in being able to go to the site to see precisely how I can do this and have a video to demonstrate just how it can be performed. So I’m interested to tap the play button.‬‬‬‬‬‬‬‬‬‬‬‬‬‬‬‬‬‬‬‬‬‬‬” [Focus Group 4, PID 1]‬‬‬‬‬‬‬ “There might be a little discussion of who might need this because if I just had RAI and I’m out of isolation, am I supposed to do this irrespective of symptoms‬?” [PID 4, Patient Advocate]‬‬‬‬‬‬‬‬	Self-efficacy and self-regulation
FAQ^c^ pageDescription: need for relevant and concise questions related to the post-RAI treatment timeframe. Stakeholders suggested providing answers to the questions within the web-based intervention to reduce the potential for increased demands on provider workflows.	“Some of the things that I would not think about are dental appointments. I didn’t think about that until recently, after I had my sialendoscopy. But other than that, all the other questions are relevant. You don’t really think about it before or during the RAI treatment, because you didn’t know that was a concern.” [Focus Group 2, PID 2]‬‬‬‬‬‬‬‬‬‬‬‬‬‬‬‬‬‬‬‬‬‬‬‬‬‬‬‬‬‬‬‬‬‬‬‬‬‬‬‬‬‬‬‬‬ “Is there a value to having answers for these [questions]? I would wonder if I’m interested in the first question, and I want to ask my provider that, I also want to know the answer now. Or I want to get some guidance about that now. I don’t want to wait until my clinic appointment in 2 weeks to get the answer. That was what, I wonder, is there a role for that? But with some answers.‬” [PID 6, Palliative Care Provider]	Self-regulation and outcome expectations

a SCT: Social Cognitive Theory.

b RAI: radioactive iodine.

c FAQ: frequently asked question.

**Table 4.** Subthemes and exemplar quotes related to domain 3 intervention content to promote RAI^a^ symptom management and survivorship care needs within the SCT^b^ framework.

Subthemes	Exemplar Quotes	SCT Construct
Nutrition description: information on nutrition strategies for RAI symptom management strategies for salivary symptoms. Topics included details about foods that stimulate salivary glands and help manage changes in taste.	“It could be a video about what foods might be helpful [to] stimulate your salivary glands. And then, in case you lost taste, recommendations for you to still taste something, and being able to eat without affecting the health of your mouth.” [Focus Group 5, PID 2] “If there were tips presented earlier about managing dry mouth like ensuring adequate hydration, ensuring fluid or moisture, rich food intake, that is something that could be helpful.” [PID 10 Registered oncology dietitian]	Self-efficacy and self-regulation
Communication with family description: information and strategies to help guide patient-led communication with families and children, about RAI symptoms. Topics included practical tips on how to talk about the social and emotional impact of DTC^c^ diagnosis and RAI symptoms with children and family members.	‪”I was thinking [what] would also be nice [is] to have some type of resource center for family and friends on how to best support someone with thyroid cancer ... [It is]a nice way of how best to support someone with thyroid cancer.” [Focus Group 4, PID 1] “‪How to speak to them, when they’re young and explain the survival rate is very good. And when it comes to cancers, it’s not [a] death sentence, because you hear the cancer part, but you know it is survivable.” [Focus Group 6, PID 3] “That’s important, especially, if your children are young, [and] they hear the diagnosis of cancer. How you can communicate that to the children, if there is information on that, I think that would be helpful for patients.” [PID 3, Nuclear Medicine Provider] “‪I worry about broad strokes because what do you tell a 6-year-old versus the 10-year-old versus a 13-year-old and not all 13-year-olds are the same.” [PID 8, Thyroid Surgeon] “‪I think that’s [communication] also intimidating for people. Some people have a better general sense of what this treatment is about, and how it can or can’t impact others. And other people are less familiar and aware or able to articulate what they know about their treatment and how it can impact others. And I think that can just be hard and an additional burden on the patient.” [PID 11, Psycho-oncologist]	Self-efficacy, and self-regulation
Cancer survivorship follow-up care description: discussed the need for a general overview of lifelong follow-up with health care providers, including, which health care team members to consult with for RAI symptom management and follow-up tests (blood, ultrasound, or whole body radioiodine scans). ‪	‪“Are you going back to the nuclear medicine team? Are you [going to] your endo? Who’s supposed to be quarterbacking all of your treatment? The right person? Is it whoever did your surgery? Who’s the right person depending on what the symptoms are?” [Focus Group 1, PID 5] “What would be helpful is how to explain to other providers that you’ve had this treatment, and these are some of the possible side effects ... I think having something like a quick reference sheet where they [patients] can talk to their doctor.” [Focus Group 6, PID 2] “Patients should know that they basically have this diagnosis of cancer, and that they need lifelong follow up. Follow up with their endocrinologists, follow up with their primary care, or whoever is managing their dose which they get for their thyroid hormone. Or to make sure that they that they do not get recurrence in the future.” [PID 3, Nuclear Medicine Provider]	Knowledge, self-regulation, and outcome expectations

a RAI: radioactive iodine.

b SCT: Social Cognitive Theory.

c DTC: differentiated thyroid cancer.

### Domain 1: Difficult-to-Manage RAI Symptoms: Short, Medium, and Late Treatment Effects

As listed in Table 5, we indicated the percentage of focus group participants and stakeholders who commented on difficult-to-manage RAI symptoms. In our sample, approximately half of our focus group participants (11/22) endorsed symptoms of dry mouth and changes in taste as challenging symptoms to manage, followed by salivary gland swelling (8/22, 36%), dry eyes (7/22, 31%), and dry nose (3/22, 13%). Stakeholders endorsed a similar prevalence of difficult-to-manage RAI symptoms experienced by patients in their clinical care, including dry mouth (6/12, 50%), changes in taste (5/12, 41%), salivary gland swelling (4/12, 33%), swallowing difficulties (3/12, 25%), and dry eyes (3/12, 25%). Focus groups highlighted that among patients with DTC post-RAI treatment, the effectiveness of their current self-management strategies varies across individuals. Participants emphasized the need for personalized approaches to manage the short and late effects of RAI treatment. Many stakeholders emphasized the need for patient referrals and collaboration with multidisciplinary care teams to enhance overall patient care and address severe RAI symptom management.

**Table 5.** RAI-specific^a^ symptoms referenced as difficult to manage by focus group participants and stakeholders during qualitative interviews.

Difficult to manage RAI symptom	Focus group participants (n=22), n (%)	Stakeholders (n=12), n (%)
**Salivary**		
Dry mouth	11 (50)	6 (50)
Change in taste	12 (55)	5 (42)
Mouth ulcers	2 (9)	2 (17)
Salivary gland swelling	8 (36)	4 (33)
Swallowing difficulties	2 (9)	3 (25)
Dental issues	4 (18)	1 (8)
**Lacrimal**		
Dry eyes	7 (32)	3 (25)
Watery eyes	—^b^	1 (8)
**Nasal**		
Dry nose	3 (14)	
Loss of smell	1 (5)	—
Nose bleeds	2 (9)	—
**Other**		
Fatigue^c^	4 (18)	4 (33)
Headache	3 (14)	—
Gastrointestinal issues	2 (9)	—
Menstrual cycle changes^c^	1 (5)	—
Hair loss	5 (23)	—
Nausea	8 (36)	3 (25)

a RAI: radioactive iodine.

b Not endorsed by any focus group participants or stakeholders.

c Symptoms may occur from concurrent treatment such as thyroid stimulating hormone therapy.

### Domain 2: Key Intervention Structure and Content Feedback on Mockups

The key message we derived from the focus group participant and stakeholder findings was that the web-based intervention requires a more streamlined home page narrative instructing patients on using the intervention in the context of experienced symptoms and personalized self-management strategies. Both focus group participants and stakeholders felt the home page mockup needed to provide relevant information about the website for a patient engaging in the intervention. Stakeholders emphasized the desirability of repeated language within the intervention content narrative to redirect patients to their health care team should emergent symptoms occur requiring medical oversight. Consequently, for the RAI support intervention to be perceived as credible, reliable, and trustworthy, focus group participants and stakeholders indicated it was very important that it include references to differentiate the intervention from nonacademic public-facing websites.

Overall, focus group participants and stakeholders found the education and self-management of symptoms mockup pages to be helpful components of a web-based intervention. Most focus group participants found that the education pages presented “distressing” symptoms in a simple and approachable visual format. Together, focus group participants and stakeholders found the RAI symptom management mockup to be useful and found the video format to allow for more engagement across a range of patient learning styles. Both patients and stakeholders emphasized the need for short video content (less than 3 minutes in length) and accessibility features such as closed captioning to maintain user video engagement. The frequently asked questions content page received mixed responses between focus group participants and stakeholders. Focus group participants highlighted the value of prepared questions to address patient RAI symptom concerns and increase patient-provider communication during appointments. In contrast, stakeholders shared concerns about the potential negative consequences on provider workload of “unanswered” questions that a patient may bring to a clinical encounter. Most stakeholders recommended answering the questions on the frequently asked questions page directly and including hyperlinks within the web-based intervention with answers to the questions. Both groups suggested the need to tailor questions to address RAI symptoms and aftercare and to use lay language to streamline information.

### Domain 3: Intervention Content to Promote RAI Symptom Management and Survivorship Care

Focus group participants and stakeholders identified topics for intervention content and characteristics that had previously not been considered in the initial intervention design. Many focus group participants expressed uncertainty about which foods to eat after RAI treatment and suggested intervention content on foods that stimulate salivary gland secretion. Stakeholders reinforced the consideration of nutrition-based self-management strategies to reduce dry mouth and changes in taste, with an emphasis on the noncurative nature of these strategies. Many focus group participants indicated recommendations on how to communicate with family members about side effects, especially in light of the “good cancer” label, would be helpful after RAI treatment. A smaller number of focus group participants with children emphasized the need for intervention content on how to communicate with children about thyroid cancer, RAI treatment-related side effects, and survivorship. Most stakeholders supported the inclusion of communication strategies with family members but emphasized tailoring communication with adolescents and children at age-appropriate levels. Focus group participants expressed challenges in long-term monitoring for the management of RAI symptoms, monitoring for potential cancer recurrence, and not knowing which provider is appropriate for RAI symptom management.

## Discussion

### Principal Results

Following the SCT framework and the person-centered approach to inform the development of a theoretically driven mHealth symptom management intervention, we elicited feedback from focus group participants and stakeholders regarding which symptoms related to RAI treatment are the most challenging to manage. Findings in this study support evidence from prior research on RAI-related toxicities [11,12,16], including well-documented salivary gland swelling, xerostomia or dry mouth, and changes in taste [15]. Both patients and stakeholders emphasized the utility of a web-based intervention for patient self-management of mild to moderate symptoms with redirection to the patient health care team to treat severe symptoms.

Findings from this study build upon prior work investigating cancer survivor self-management interventions [44,45] with an emphasis on patient knowledge, self-efficacy, and skill development as intervention components from SCT [30,46]. Most self-management interventions are designed for a particular type of cancer or symptom [44,46], which does not capture the RAI treatment aftercare experiences among patients with DTC. RAI treatment-specific issues and aftercare needs reported by our focus group participants and stakeholders reinforce prior evidence of unmet informational needs related to RAI side effects and long-term symptom management in this population [1,17,19,47]. Both focus group participants and stakeholders found the content and digital format of the web-based mockups of salivary symptom education and a salivary gland massage video helpful in engaging and empowering patients in symptom self-management. These findings support the potential of web-based symptom management interventions in patient outcomes with accessible and digital information and support [20,28,44].

Additionally, our results may potentially address supportive care gaps through focus group patient and stakeholder identification of nutrition management strategies, communication with family, and follow-up care planning as tailored survivorship components to include in a web-based intervention. Survivorship care among this patient population involves unique coordination across specialists, with an endocrinologist or primary care provider at the center [17,47], and adjunct supportive programs such as a web-based intervention for patient self-management of RAI symptoms may offer assistance in reducing fragmented care gaps, improving self-efficacy in RAI symptom management, and reducing patient health care utilization.

### Limitations

There are several limitations to this study. Our patients and stakeholders primarily identified as White and non-Hispanic, which limits the generalizability of the data. Furthermore, our patient sample comprised insured patients with thyroid cancer, and our qualitative findings did not capture the perspective of uninsured patients. Our qualitative results identified a gap in the current delivery of coordinated patient care across subspecialties. It should be noted that this care gap is reflective of one academic hospital center and is not generalizable across all settings. While our results reached saturation across stakeholder specialists, saturation within subspecialists did not occur due to the limited number of subspecialists enrolled in each group. In addition, we were not able to engage a dentist as a stakeholder. Future qualitative research should elicit endocrinologists’ and primary care providers’ perspectives on mHealth behavioral interventions for the self-management of RAI symptoms to enhance the implementation of coordinated care efforts.

A small subset of focus group participants attributed fatigue and menstrual cycle changes to RAI treatment; however, these are symptoms from concurrent treatments such as thyroidectomy and thyroid-stimulating hormones. Patients may have difficulty differentiating which symptoms are due to RAI or other concurrent treatment. In addition, symptoms such as menstrual cycle changes could be due to both RAI and adjustment of thyroid hormone dosage. Future studies can work toward disambiguating symptoms for patient education and content development of a web-based intervention for RAI treatment symptom management.

### Conclusions

Through a SCT framework and person-based approach, results from this qualitative study highlight important intervention content considerations such as the inclusion of nutrition management strategies, communication skills, and follow-up care planning for the self-management of RAI symptoms among patients with DTC. These findings can inform the development of future self-management interventions tailored to patients with DTC after RAI treatment.

## Supplementary material

10.2196/60588 Multimedia Appendix 1 The open-ended qualitative focus group moderator guide is informed by Social Cognitive Theory and person-based design principles to elicit patient perspectives about RAI symptoms that were challenging to manage, identify content that would be helpful in a symptom management website, and obtain feedback on the formatting and content of static images or mockups.

10.2196/60588 Multimedia Appendix 2 The open-ended qualitative semistructured interview guide is informed by Social Cognitive Theory and person-based design principles to elicit stakeholder perspectives about RAI symptoms that are challenging to manage, identify content that would be helpful in a symptom management website, and obtain feedback on the formatting and content of static images or mockups.

10.2196/60588 Multimedia Appendix 3 Four mockups of a prospective web-based radioactive iodine treatment symptom management intervention reviewed by focus group participants and stakeholders: 1) home page, 2) salivary gland massage video, 3) salivary gland education page, and 4) frequently asked questions page.

## Abbreviations

COREQ: Consolidated Criteria for Reporting Qualitative Research
DTC: differentiated thyroid cancer
HRQOL: health-related quality of life
mHealth: mobile health
RAI: radioactive iodine
REDCap: Research Electronic Data Capture
SCT: Social Cognitive Theory
