# Supplementary material for: Patient and Provider Perspectives of a Web-Based Intervention to Support Symptom Management After Radioactive Iodine Treatment for Differentiated Thyroid Cancer: Qualitative Study
Source: JMIR Form Res. 2025 Mar 19;9:e60588. doi: 10.2196/60588 (PMC11939019; doi:10.2196/60588)
Supplement: Multimedia Appendix 1 [file formative-v9-e60588-s001.docx]

**Focus Group Moderator’s Guide**

**Section 1: Start Group (10 minutes)**

*Thank you for being here. We want to welcome you today and share our appreciation for your time and willingness to participate. My name is* **_____** *and today I will be leading our focus group discussion. A focus group is a meeting where questions are asked to a group of people about a specific topic. Researchers use focus groups to learn about a topic from people who have first-hand experience with it. The purpose of this focus group is for us to listen to your thoughts about the types of information, tools and resources that would be useful for patients with thyroid cancer to manage symptoms related to radioactive iodine treatment. We will abbreviate radioactive iodine treatment as RAI during this discussion. We are specifically interested in your thoughts about the content and visual appeal of a future informational support website.*

*You should feel free to make any sort of comments – positive or negative – about what we are talking about today. There are no right or wrong answers. We truly appreciate your help. We are interested in finding ways to help support patients managing symptoms related to RAI and all experiences are valuable as we learn more about this process.*

*1.* The moderator will briefly remind participants of key sections of the consent form and answer questions.

*We emailed you a consent form to read and sign and received it back from all of you, along with a brief survey. Right now, I’d like to take a moment to review the main points of the consent form and answer any questions you have. Remember that your participation is completely voluntary and you can decide you want to stop participating at any time.*

Important points to note:

- Protection of privacy
  - Be sure to note that today’s discussion will be recorded. If you do not feel comfortable with this, please send a private chat message to [STAFF MEMBER]. As a reminder, your name will never be associated with your responses.
  - You can choose to skip any questions you do not wish to answer; you can simply say “pass” if we ask for your input you and you do not wish to answer.
- Who to contact with questions after the group is over.

*Are there any questions?*

2. The moderator will review the ground rules.

*We want everyone to have the chance to share their opinions or experiences. We hope that this will be a very open discussion. There are just a few ground rules we want to go over that will help everything go more smoothly:*

- *Talk one at a time.*
- *Be respectful of others. You don’t have to agree with what’s said.*
- *Keep today’s discussions private. What is said in this videoconference should stay here.*
- *If you do not want to answer one or more questions, that’s okay. You don’t have to and as we shared earlier, you can simply say “pass.”*
- *Please keep your camera on if you feel comfortable, and mute when you are not talking to reduce background noise.*
- *Feel free to use the zoom ‘reaction’ emojis if you wish (located under the reactions button at the bottom of the page).*
- *Please do not take any screen shots to protect people’s privacy.*
- *We may call on people to make sure we hear different opinions and perspectives so please make sure you are in front of your computer ready to participate. If you need to step away for a moment during this meeting, please let us know by speaking up or sending a message to a study team.*
- *Does anyone have any questions? Great. Now I’ll start recording.*

**(START RECORDING)**

**Section 1: Introduction/Opening Discussion (20 minutes)**

1. Ask them to introduce themselves, first name only or pseudonym if they prefer.

1. *Let me start by asking some general questions.* *Think back to when you had RAI treatment -- was there anything that took you by surprise or that you wished you had known about ahead of time?*

**Section 2: Symptom Burden and Support (35 minutes)**

*Let’s talk about the symptoms you experienced from RAI.*

1. *What symptoms were the most challenging to manage after RAI?*
   1. *Describe the ways you managed these symptoms.*
   2. *Probe: Short-term symptoms (e.g., nausea and vomiting, dry mouth, taste changes, neck tenderness and swelling, swelling and tenderness of the salivary glands)*
   3. *Probe: Long-term symptoms (e.g., tiredness, inflammation of salivary glands, dry or watery eyes)*
2. *Are there symptoms that you are still struggling with that have yet to be resolved?*
3. *Did you seek out information on your own about RAI symptom management?*
   1. *Where did you seek out information from?*
      1. *Probe: healthcare team, online information, Thyroid cancer support groups/advocacy groups, other people who had been diagnosed with thyroid cancer?*
   2. *What information did you find that was helpful?*
4. *At any point in your RAI treatment, did you feel you needed more information on how to manage treatment related symptoms?*
   - 1. *If yes, what information was missing?*
     2. *If yes, what else did you need at the time?*
5. *During and after your RAI treatment were there any resources or people that provided support that were particularly helpful in managing stress, worry or other emotions?*
   1. *Were there times you felt you needed more support in times of stress or other emotions?*
   2. *If yes, did you seek additional support?*
   3. *Where did you tend to go when you needed more support?*
6. *From whom (probe: support group, friends/family support, psychologist, counselor, etc.)?*
7. *In what format (in-person, online)?*

**Section 3: Website Topics and Format (35 minutes)**

*We are interested in developing a website to help support patients with thyroid cancer manage symptoms related to RAI. We are thinking of including information on the website about different topics.*

1. *What topics come to mind when you think of a website with information on RAI symptom management?*
2. *How would you feel about the website including patient testimonial videos?*
3. *For example, patients sharing their experience managing symptoms after RAI treatment? What would you want to see or not see in patient testimonials? What could help patient testimonial videos feel relevant to you?*
4. *What are your thoughts on including resources for adjusting to cancer and treatment?*
5. *For example, information on how to talk to loved ones about treatment-related symptoms? What would you want to see or not see in resources for adjusting to cancer and treatment?*
6. *What are your thoughts on including self-guided relaxation audio recordings to help patients with worry or anxious thoughts about their cancer diagnosis, treatment or long-term well-being? What would you want to see or not see in self-guided relaxation recordings?*
7. *People have different preferences for how to receive health information. Some people prefer visual information when receiving health information. Whereas other people prefer numbers when receiving health information. What is your style of preference?*

**Instructions:** Moderator shares their screen on Zoom to display the iSupport homepage Mockup.

*Patients from our previous studies indicated general information related to RAI and differentiated thyroid cancer would be helpful to include on the website. Differentiated thyroid cancer is the type of thyroid cancer that may be treated with RAI. We created a mockup of the website homepage with an overview of how many people are diagnosed each year with thyroid cancer and how many newly diagnosed patients with thyroid cancer receive RAI.*

1. *What are your thoughts on this mock up?*
   1. *What questions come to mind as you look at it?*
   2. *If a patient were to look at the homepage in its current format, is there anything that would make this hard to understand?*
   3. *Is there anything that would make this easier to understand?*
2. *Can you think of other information that would be useful to include on this page?*
3. *What are your thoughts on the name of the website: iSupport (informational support for patients post RAI treatment)? What are your thoughts on the color scheme, layout, and logo?*

**Instructions:** Moderator shares their screen on Zoom to display the How to Massage Salivary Glands Video Mockup

*Patients from prior studies indicated that clinician-approved short symptom management educational videos would be helpful to include on the website. We created a mockup of a short (less than 4 minute) video guided activity of how to massage salivary glands.*

1. *What are your thoughts on including a video guided activity like this one?*
   1. *How long do you think the video guided activities should be? What would be too long to hold your attention?*
      1. *Would anything get in the way of using the video?*
         1. *Probe: Pace of instructions*
         2. *Probe: Complex medical jargon*
         3. *Probe: Preference for video with a transcript included versus images with text description*
         4. *Probe: Video activity led by a patient who seemed very different than me*
2. *What would make this easier to use in its current format?*
3. *What would make this harder to use in its current format?*
4. *If you had access to video guided activities for symptom management, what types of videos would you want to watch?*
   1. *What types of video guided activities would you not want to watch, or would not be helpful?*
      1. *Probe: Why?*

**Instructions:** Moderator shares their screen on Zoom to display the Common Salivary Side Effects Mockup.

*Patients in our previous studies recommended including a list of common symptoms from RAI treatment and also including uncommon symptoms you should discuss with your health care provider. We put together a mockup of what that list could look like.*

1. *What are your thoughts on the content and layout of this list?*
2. *What feels like it is missing from this list?*
   1. *Are there any symptoms, common or uncommon, that are missing from this list?*

**Instructions:** Moderator shares their screen on Zoom to display the iSupport Patient Cheat Sheet Mockup.

*We’ve heard from folks in our previous studies that prompts/reminder questions would be helpful information to have on the website. We put together a mockup of what that list could look like. You’ll see here 6 main types of questions.*

*What are your thoughts on these questions?*

*What are your thoughts on the layout of this page?*

*What might be missing from this list?*

1. *As a reminder - no one here has to share any personal medical information. Are there any example questions you are happy you asked before?*
2. *Or in hindsight are there questions that you wish you would have asked?*
3. *Is there anything on this list you wouldn’t want on this list?*
4. *Is there anything on this list you wouldn’t want to be printed out?*

*Now that you’ve seen the mockups of the informational support website for symptom management, what would keep you coming back to a website like this?*

1. *What would get you to return to the site for more information?*

**Section: Closing (5 minutes)**

*Based on our discussion today, what do you feel are two main points I should take back to our team?*

*Is there anything else you feel we did not cover that we need to know?*

*We would like to thank you for your time.  Your answers have greatly helped us.  If you have any questions about what we have done today, do not hesitate to call the phone number on the bottom of the consent form.  We will be happy to talk with you about the study. Also, if anyone would like a summary of results from the focus group, we would be happy to provide a summary. We will make sure to take out anyone’s name or other identifying information. It will be a few months before the summary results are ready, but we are happy to share with anyone who is interested. You can* email [*study email address*] *to indicate interest or to ask any follow up questions [post email address in the chat].*

**(STOP THE RECORDER)**

**[Discuss distribution of incentives and paperwork]**
