# Supplementary material for: Patient and Provider Perspectives of a Web-Based Intervention to Support Symptom Management After Radioactive Iodine Treatment for Differentiated Thyroid Cancer: Qualitative Study
Source: JMIR Form Res. 2025 Mar 19;9:e60588. doi: 10.2196/60588 (PMC11939019; doi:10.2196/60588)
Supplement: Multimedia Appendix 2 [file formative-v9-e60588-s002.docx]

**Individual Stakeholder Semi-Structured Interview Guide**

**Section 1: Symptom Burden and Support (15 minutes)**

Instructions: The interview will ask providers, including endocrine surgeons, nuclear medicine providers, endocrinologists, palliative care providers, social workers, psycho-oncologists, and registered dieticians, the questions below the provider prompts.

Provider prompts:

*Let’s talk about the symptoms your patients experience from RAI.*

1. *What patient symptoms are the most challenging to manage after RAI?*
   1. *Describe the ways your patients managed these symptoms.*

Instructions: The interviewer shares “Over the counter product list” on the video conference platform.

*
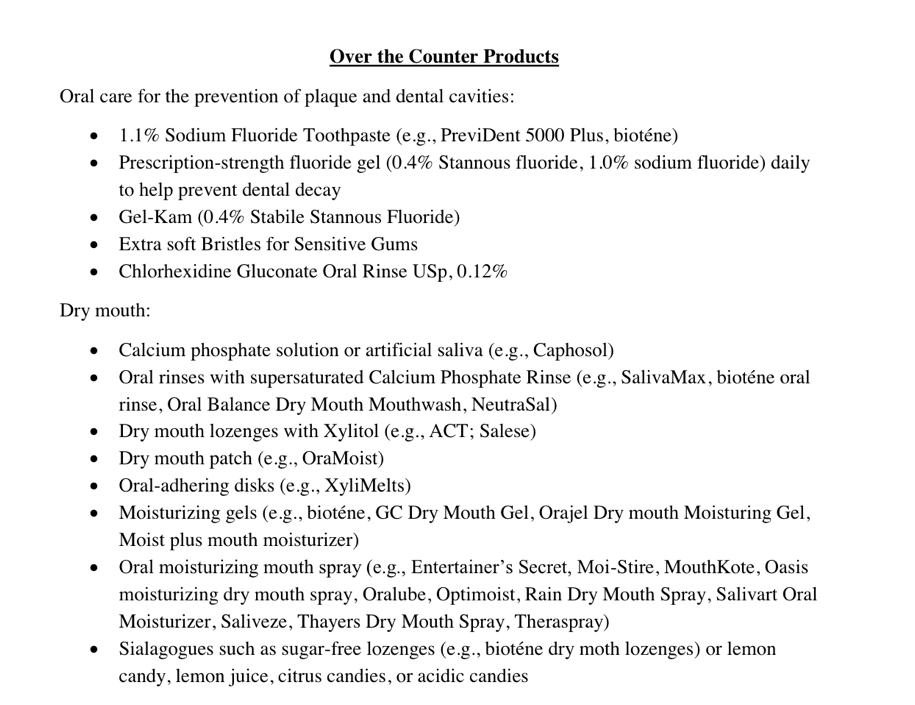
*

1. *I am going to show you a list of over-the-counter products and medications recommended by medical specialty associations for the management of symptoms related to RAI treatment. Please read the list and say out loud any over-the-counter medications or products that you routinely recommend to patients for RAI symptom management.*
   1. *Probe: Over-the-counter medications for short-term symptoms*
   2. *Probe: Over-the-counter medications for long-term symptoms*
   3. *Probe: Are there any over-the-counter medications missing from this list?*
2. *Are there symptoms that the patients you provide care for tend to still struggle with that have yet to be resolved?*

Instructions: The interview will ask patient advocates the questions below the patient advocate prompts.

Patient advocate prompts:

*Let’s talk about the symptoms you often see individuals with differentiated thyroid cancer experience from RAI.*

1. *What symptoms are the most challenging to manage after RAI?*
   1. *Describe the ways you’ve heard members of ThyCa manage these symptoms.*

Instructions: The interviewer shares “Over the counter product list” on the video conference platform.

*
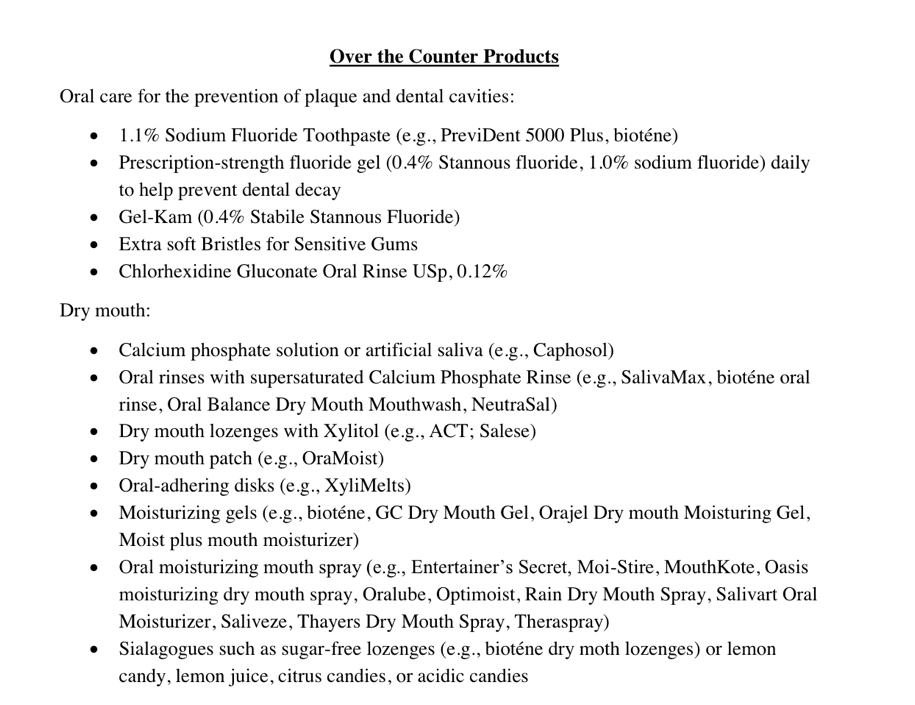
*

1. *I am going to show you a list of over-the-counter products and medications recommended by medical specialty associations for the management of symptoms related to RAI treatment. Please read the list and say out loud any over-the-counter medications or products that ThyCa members routinely recommend for RAI symptom management.*
   1. *Probe: Over-the-counter medications for short-term symptoms*
   2. *Probe: Over-the-counter medications for long-term symptoms*
   3. *Probe: Are there any over-the-counter medications missing from this list?*
2. *Are there symptoms that the ThyCa members you work with tend to still struggle with that have yet to be resolved?*

**Section 2: Website Topics and Format (35 minutes)**

*We are interested in developing a website to help support patients with thyroid cancer to manage symptoms related to RAI. Based on our focus group discussions with patients, they identified the following topics to include on the website:*

1. *A general overview of symptom management. For example, information on over-the-counter products to manage symptoms such as dry mouth. How would you feel about the website containing this topic?*
   1. *Probe: Pros.*
   2. *Probe: Cons. What would be problematic with information on over-the-counter products?*
2. *RAI dose and side effects. For example, statistics or frequencies of anticipated side effects based on RAI dose received. What would you want to see or not see in RAI dose and side effects on the website?*
3. *How to cope with immediate side effects from RAI. For example, a patient testimonial video describing how a patient coped and managed immediate side effects from RAI. What would you want to see or not see in the patient testimonial videos?*
4. *Tell me about the type of information you would find helpful related to nutrition following RAI treatment.*
   1. *Probe: For example, some patients in our focus groups indicated that information on foods that stimulate the salivary glands or foods that promote dental health might be useful. Please share your thoughts.*
5. *A resources page. For example, information on how to talk to loved ones about treatment-related symptoms. Please share your thoughts.*
   1. *What would you want to see or not see in resources on how to communicate to loved ones about treatment-related symptoms?*
6. *A resources page. For example, information on how to find a mental health specialist such as a psychologist, social worker, or counselor?*
7. *What are your thoughts on including resources for adjusting to cancer and treatment? For example, self-guided relaxation audio recordings to help patients with worry or anxious thoughts about their cancer diagnosis, treatment or long-term well-being?*
8. *What other topics come to mind when you think of a website with information on RAI symptom management?*
   1. *How would you feel about a provider testimonial video of commonly asked questions?*
9. *People have different preferences for how to receive health information. Some people prefer visual information when receiving health information. Whereas other people prefer numbers when receiving health information. What style of preference would be most beneficial for this patient population and web-based platform?*
10. *At what point in the RAI treatment process would patients most benefit from a website to help support patients with thyroid cancer to manage symptoms related to RAI?*

**Instructions:** Interviewer shares their screen on Zoom to display the iSupport homepage Mockup.

*Patients from our previous studies indicated that general information related to RAI and differentiated thyroid cancer would be helpful to include on the website. We created a mockup of the website homepage with an overview of how many people are diagnosed each year with thyroid cancer and how many newly diagnosed patients with thyroid cancer receive RAI.*

1. *We thought we would start with general information patients in our focus group study provided about the homepage. Overall, patients from our focus group stated that they liked the statistics on the homepage but felt the information about the incidence rate was something they would already know. What are your thoughts on this mock up?*
2. *Is there any value in having statistics about RAI front and center?*
3. *Can you think of other information that would be useful to include on this page?*
4. *Focus group patients indicated that they felt the name of the website iSupport (informational support for patients post RAI treatment) was either too vague or implied support of RAI. What are your thoughts on the name of the website: iSupport (informational support for patients post RAI treatment)?*
   1. *What are your thoughts on alternative names of the website such as Informed after RAI? Or Re-inform? IARAI… Informed After RAI?*
5. *What are your thoughts on the color scheme, layout, and logo?*

**Instructions:** Interviewer shares their screen on Zoom to display the How to Massage Salivary Glands Video Mockup

*Patients from prior studies indicated that clinician-approved short symptom management educational videos would be helpful to include on the website. We created a mockup of a short (less than 4 minute) video guided activity of how to massage salivary glands.*

1. *Overall, participants from our focus group discussions indicated they liked the topics “How to Massage Salivary Glands.” They also indicated that engagement in this video would depend on whether they were experiencing salivary gland swelling. What are your thoughts on including a video guided activity like this one?*
2. *Patient advocates only: Patients from our focus groups indicated that the use of closed captioning or video transcripts would make the video easier to use. What else would make this easier to use in its current format?*
3. *As a provider or patient advocate, what videos do you use for patient education as a tool?*
   1. *Prompt: What makes it easier for patients to use videos for education purposes?*
4. *As a provider or patient advocate what types of symptom management videos would you recommend to patients to watch?*
   1. *As a provider or patient advocate, what types of video-guided activities would you not recommend to patients or would not be helpful?*
      1. *Probe: Why?*
   2. *What other types of “how-to videos” for symptom management would be helpful?*

**Instructions:** Interviewer shares their screen on Zoom to display the Common Salivary Side Effects Mockup.

*Patients in our previous studies recommended including a list of common symptoms from RAI treatment and also including uncommon symptoms you should discuss with your health care provider. We put together a mockup of what that list could look like.*

1. *What are your thoughts on the content and layout of this list?*
2. *How accurate are the salivary gland symptoms listed in this mockup?*
3. *What feels like it is missing from this list?*
   1. *Are there any symptoms, common or uncommon, that are missing from this list?*

**Instructions:** Interviewer shares their screen on Zoom to display the iSupport Patient Cheat Sheet Mockup.

*We’ve heard from folks in our previous studies that prompts/reminder questions would be helpful information to have on the website. Participants from our focus groups recommended incorporating an interactive feature where they could check off the relevant questions to bring with them to medical appointments. We put together a revised mockup of what that list could look like. You’ll see here 8 main types of questions What are your thoughts on these questions?*

*What might be missing from this list?*

1. *Is there anything on this list you wouldn’t want on this list?*
2. *Is there anything on this list you wouldn’t want to be printed out?*

Patient advocate prompt only:

*Now that you’ve seen the mockups of the informational support website for symptom management, what would keep a patient coming back to a website like this?*

1. *What would get a patient to return to the site for more information?*

**Section: Closing (5 minutes)**

*Based on our discussion today, what do you feel are two main points I should take back to our team?*

*Is there anything else you feel we did not cover that we need to know?*
