# Supplementary material for: Patient and Provider Perspectives of a Web-Based Intervention to Support Symptom Management After Radioactive Iodine Treatment for Differentiated Thyroid Cancer: Qualitative Study
Source: JMIR Form Res. 2025 Mar 19;9:e60588. doi: 10.2196/60588 (PMC11939019; doi:10.2196/60588)
Supplement: Multimedia Appendix 3 [file formative-v9-e60588-s003.pdf]

44,280 PEOPLE ARE EXPECTED TO BE DIAGNOSED WITH DIFFERENTIATED THYROID CANCER (DTC) THIS YEAR

Radioactive iodine treatment (RAI) is routinely used for patients with DTC, with up to 76% of patients with DTC receiving RAI - SEER, 2021

CLICK HERE TO LEARN MORE ABOUT RAI SPECIFIC SIDE EFFECTS

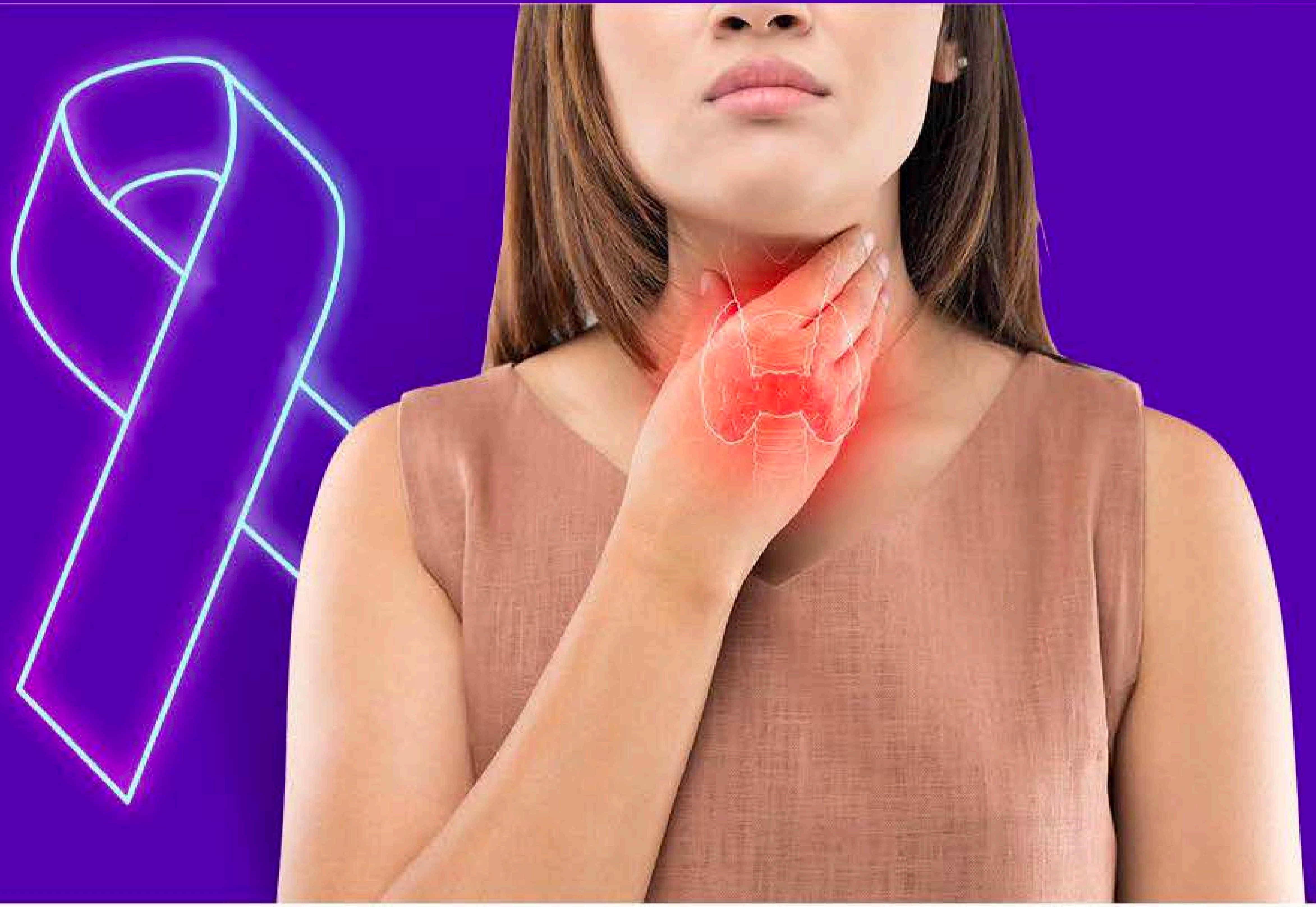

HOW TO MASSAGE SALIVARY GLANDS

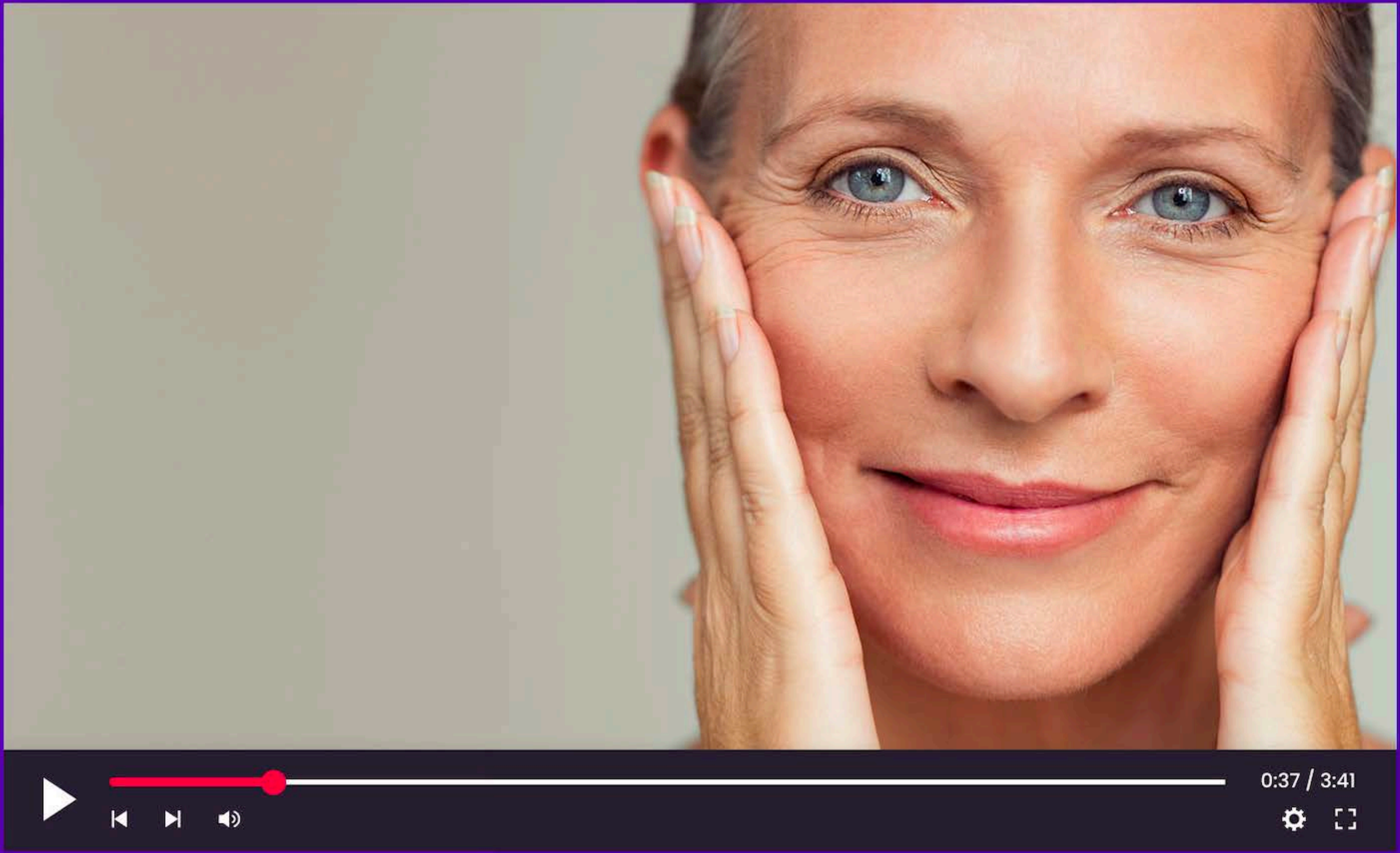

COMMON SALIVARY SIDE EFFECTS FROM RAI

- **Dry Mouth (Xerostomia)**
- **Plaque**
- **Bad Breath (Halitosis)**
- **Difficulty eating and swallowing dry food**
- **Pain or Soreness on the Face**
- **Facial Swelling**
- **Metallic Taste**
- **Lack of taste**

SEVERE SALIVARY SIDE EFFECTS TO DISCUSS WITH YOUR HEALTH CARE PROVIDER

If you develop any of these salivary side effects, please call your health provider immediately and seek medication attention.

- **Redness over salivary glands**
- **Pain doesn’t go away with anti-inflammatory medications**
- **Worsening swelling of salivary glands- sign of infection**
- **Significant weight loss due to difficulty eating and swallowing**

- Mohammed, A. A. (2014). Update knowledge of dry mouth-A guideline for dentists. African health sciences, 14(3), 736-742.
- Dr. Venetia Aranha, Dr. Meghana S.M., Dr. Monica Yadav, Dr. Jashika Shroff. (2020). Xerostomia in patients undergoing anticancer therapy. World Journal of Advanced Scientific Research, 3(5), 1 - 15. Retrieved from https://ubipayroll.com/wjasr/index.php/wjasr/article/view/175

PATIENT CHEAT SHEET TO ASK PROVIDERS

Check the boxes below to create a personalized list of questions to ask your provider.

- [x] After receiving radioactive iodine treatment, what should I anticipate in terms of next steps for treatment or monitoring?
- [x] I am experiencing difficulties swallowing and these difficulties are making me choke when I eat. What do you recommend to help with this problem?
- [x] Does radioactive iodine treatment interfere with adjustments being made in my thyroid hormone to get my TSH levels where they should be?
- [x] How often should I schedule dental appointments after my radioactive iodine treatment?
- [x] When I experience salivary gland side effects from radioactive iodine treatment, should I reach out to my endocrinologist, ear nose, and throat (ENT) provider, or nuclear medicine provider?
- [x] What treatments or medications may reduce the risk of developing oral inflammation?
- [x] How common is it for patients with differentiated thyroid cancer to have more than one course of radioactive iodine treatment? Is radioactive iodine treatment effective for all thyroid cancer types?
- [x] What are potential severe side effects of radioactive iodine treatment that may be an emergency?
